# Domain agnostic online semantic segmentation for multi-dimensional time series

**DOI:** 10.1007/s10618-018-0589-3

**Published:** 2018-09-25

**Authors:** Shaghayegh Gharghabi, Chin-Chia Michael Yeh, Yifei Ding, Wei Ding, Paul Hibbing, Samuel LaMunion, Andrew Kaplan, Scott E. Crouter, Eamonn Keogh

**Affiliations:** 1 Department of Computer Science and Engineering, University of California, Riverside, USA; 2 Department of Computer Science, University of Massachusetts Boston, Boston, USA; 3 Department of Kinesiology, Recreation, and Sport Studies, The University of Tennessee Knoxville, Knoxville, USA

**Keywords:** Time series, Semantic segmentation, Online algorithms

## Abstract

Unsupervised semantic segmentation in the time series domain is a much studied problem due to its potential to detect unexpected regularities and regimes in poorly understood data. However, the current techniques have several shortcomings, which have limited the adoption of time series semantic segmentation beyond academic settings for four primary reasons. First, most methods require setting/learning many parameters and thus may have problems generalizing to novel situations. Second, most methods implicitly assume that all the data is segmentable and have difficulty when that assumption is unwarranted. Thirdly, many algorithms are only defined for the single dimensional case, despite the ubiquity of multi-dimensional data. Finally, most research efforts have been confined to the batch case, but online segmentation is clearly more useful and actionable. To address these issues, we present a multi-dimensional algorithm, which is domain agnostic, has only one, easily-determined parameter, and can handle data streaming at a high rate. In this context, we test the algorithm on the largest and most diverse collection of time series datasets ever considered for this task and demonstrate the algorithm’s superiority over current solutions.

## Introduction

The ubiquity of sensors and the plunging cost of storage has resulted in increasing amounts of time series data being captured. One of the most basic analyses one can perform on such data is to segment it into homogenous regions. We note that the word “segmentation” is somewhat overloaded in the literature. It can refer to the approximation of a signal with piecewise polynomials (Keogh et al. 2004), or the division of a time series into internally consistent regimes. For clarity, this latter task is sometimes called “semantic segmentation” (Yeh et al. 2016; Aminikhanghahi and Cook 2017), where there is no danger of confusion, and we will simply refer to it as segmentation. It can, at times, be fruitful to see segmentation as a special type of clustering with the additional constraint that the elements in each cluster are contiguous in time.

The utility of segmentation is myriad. For example, if one can segment a long-time series into *k* regions (where *k* is a small), then it may be sufficient to show only *k* short representative patterns to a human or a machine annotator in order to produce labels for the entire dataset. As an exploratory tool, sometimes we can find unexpected and actionable regularities in our data.

**Fig. 1:**
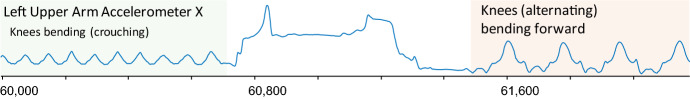
A snippet of time series collected during an exercise routine. Both the first and last third are well-defined motions, but the section in the middle is less structured, representing a transition between apparatuses

While there are many techniques for segmentation (Lainscsek et al. 2013; Reinhardt et al. 2013; Matsubara et al. 2014a; Lin et al. 2016; Aminikhanghahi and Cook 2017), they all have one or more limitations that have prevented their utilization in real world settings. This observation has motivated us to introduce FLOSS (Fast Low-cost Online Semantic Segmentation), a novel algorithm which, to the best of our knowledge, is unique in offering all the following features:

- *Domain Agnosticism* Most techniques in the literature are implicitly or explicitly suited to a single domain, including motion capture (Lan and Sun 2015; Aminikhanghahi and Cook 2017), motion capture of *upper*-*body* only (Aoki et al. 2016), electroencephalography (Kozey-Keadle et al. 2011), music (Serra et al. 2014), automobile trajectories (Harguess and Aggarwal 2009), or electrical power demand (Reinhardt et al. 2013). For example, the detailed survey in (Lin et al. 2016) notes that for almost all methods “some prior knowledge of the nature of the motion is required.” In contrast, FLOSS is a domain agnostic technique that makes essentially no assumptions about the data.
- *Streaming* Many segmentation algorithms are only defined for batch data (Lainscsek et al. 2013; Aminikhanghahi and Cook 2017). However, a streaming segmentation may provide *actionable* real-time information. For example, it could allow a medical intervention (Weiner and Charles 1997; Mohammadian et al. 2014), or a preemptive repair to a machine that has entered a failure mode (Molina et al. 2009). We will demonstrate that our FLOSS algorithm is fast enough to ingest data at 100 Hz (a typical rate for most medical devices/accelerometers) without using more than 1% of the computational resources of a typical desktop machine.
- *Real World Data Suitability* Most techniques assume that *every* region of the data belongs to a well-defined semantic segment. However, that may not be the case. Consider data from an accelerometer worn on the wrist by an athlete working out at a gym. Examined at the scale of tens of seconds, there will be many well-defined homogenous regions of behavior, corresponding to various repetitions on the apparatus (see Fig. 1). However, it is probable that there are many minutes of behavior that accumulated while the athlete was waiting her turn to use a machine. These periods may be devoid of obvious structure. Any model that insists on attempting to explain *all* of the data may be condemned to poor results. In contrast, FLOSS can effectively ignore these difficult sections.

Beyond introducing the FLOSS algorithm, we claim the following contributions to the literature:

- Most research efforts in this domain test on limited datasets (Lainscsek et al. 2013; Aminikhanghahi and Cook 2017). The authors of (Matsubara et al. 2014a) and (Zhao and Itti 2016) are both to be commended for considering *three* datasets, but they are exceptional, considering *one* dataset is the norm. In contrast, we test on a data repository of thirty-two datasets from diverse domains, *in addition* to datasets from five detailed cases studies. We believe that this free public archive will accelerate progress in this area, just as the TREC datasets have done for text retrieval, and the UCR archive has done for time series *classification* (Chen et al.).
- While classification, clustering, compression etc. all have formal and universally accepted metrics to assess progress and allow meaningful comparison of rival methods, the evaluation of segmentation algorithms has often been anecdotal (Lin et al. 2016). Evaluation is often reduced to the authors asking us to visually compare the output of their algorithm with the ground truth. While there is nothing wrong with visually compelling examples or anecdotes, it is clearly desirable to have more formal metrics. In (Matsubara et al. 2014a), the authors adapt precision/recall, but in some contexts, this is unsuitable for semantic segmentation. In Sect. 3.6, we introduce a metric that allows us to meaningfully score segmentations given some external ground truth.

We must qualify our claim that FLOSS requires only a single parameter. We note that while the *segmentation* really does require only a single parameter, the regimen extraction steps do require two additional, but inconsequent parameters. In addition, the option to add domain knowledge also requires a parameter. Nevertheless, in any sense, our algorithm is truly parameter-lite.

The rest of this paper is organized as follows. In Sect. 2, a summary of the background and related work, along with the necessary definitions, is provided. In Sect. 3.1, a batch algorithm for semantic segmentation before generalizing it to the streaming case is introduced. Section 4 illuminates a detailed quantitative and qualitative evaluation of our ideas. Finally, in Sect. 5, conclusions and directions for future work are offered.

## Background and related work

In this section, we introduce all the necessary definitions and notations and consider related work. Because the term “segmentation” is so overloaded in data mining, even in the limited context of time series, we also explicitly state what we are *not* attempting to do in this work.

Note that for clarity and brevity, our definitions and algorithms in this section only consider the one-dimensional cases; however, the generalizations to the multi-dimensional case are trivial and are explained in Sect. 3.4 (Keogh 2017).

### Definitions

Here we introduce the necessary definitions and terminology, beginning with the definition of a time series:

#### **Definition 1**

A time series ***T***=* t*_*1*_*, t*_*2*_*, t*_*3*_*, …,t*_*n*_ is a contagious, ordered sequence of real values in equally spaced time intervals of length *n*.

Our segmentation algorithm will exploit the similarity of local patterns within ***T***, called *subsequences*:

#### **Definition 2**

A *subsequence****T***_*i,L*_ of a ***T*** is a subset of the values from ***T*** of length *L* starting from position *i*. ***T***_*i,L*_=* t*_*i*_*, t*_*i*+*1*_*,…, t*_*i*+*L*-*1*_, where 1 ≤ *i* ≤ *n*-*L *+ 1.

The time series ***T*** is ultimately recorded because it is (perhaps indirectly) measuring some aspect of a system ***S*** (perhaps *indirectly* measuring the phenomenon in some instances).

#### **Definition 3**

A system ***S*** is a physical or logical process containing two or more discrete states separated by one or more boundaries ***b***.

We further explain and justify our assumption that ***S*** can be considered intrinsically discrete in Sect. 3.

The algorithms we present are built on the recently introduced Matrix Profile (MP) representation, as well as the STAMP and STAMP*I* (the online variation) algorithms used to compute it (Yeh et al. 2016). We briefly review these in the next section.

### Matrix profile background

STAMP is an all-pairs, one-nearest-neighbor search algorithm for time series (also known as *similarity join*) that leverages the Fast Fourier Transform for speed and scalability. The input parameters are the time series data ***T*** and a subsequence length *L*, where *L* is the desired length of the time series pattern to search for. For output, it returns two vectors, *MPValues* and *MPIndex*, both of which are the same length of ***T*** and can be seen as annotating it. At the index *i* of the data structure…

- *MPValues*, is the Euclidean distance of the subsequence ***T***_*i,i*+*L*_ to its nearest neighbor elsewhere in ***T***. To prevent trivial matches where the subsequence matches to itself, an exclusion region is enforced, such that the distance between ***T***_*i:i*+*L*_ and any subsequence beginning at [*i* - *L*/2: *i* + *L*/2] is assumed to be *infinity*.
- *MPIndex*, is the location of *i*’s nearest neighbor in ***T***. Note that in general, this nearest neighbor information is not symmetric, *i*’s nearest neighbor may be *j*, but *j*’s nearest neighbor may be *k*.

This review is necessarily brief, so we refer the reader to the original paper for more details (Yeh et al. 2016).

### What FLOSS is not

Even within the narrow context of time series analytics, the term *segmentation* is overloaded; thus, it is necessary to explicitly explain some tasks we are *not* addressing.

*Change point detection* is a method for detecting various changes in statistical properties of time series, such as the mean, variance or spectral density. A helpful review of the literature on this problem is surveyed in detail in a recent paper (Aminikhanghahi and Cook 2017). In contrast to change point detection, we are interested in regimens which are defined by changes in the *shapes* of the time series subsequences, which can change without any obvious effect on the *statistical* properties. Consider the following pathological example. Suppose we took an hour of an normal electrocardiogram, and appended to it a *reversed* copy of itself (to be clear, the discrete analogue of this is the production of the palindrome..beatbeatbeat*taebtaebtaeb*..). While such a time series would have a visually obvious (indeed, *jarring*) transition at the halfway point, virtually all change point algorithms that we are aware of would ignore this transition, as the features they consider (mean, standard deviation, zero-crossings, autocorrelation etc.) are invariant to the direction of time. Clearly, one can also create pathological datasets that would stymie our proposed algorithm but be trivial for most change detection algorithms. In other words, they are only superficially similar tasks that do not directly inform each other.

Similar to the stated goals, recent work on change point detection has begun to stress the need to be parameter-free and have few assumptions (Matteson and James 2014). However, scalability is rarely a priority; therefore, a typical dataset considered in this domain is a few hundred data points. This suggests that human inspection is often a competitive algorithm. However, due to the scale of the data we wish to consider and the necessity to detect regime changes where they would be difficult to discern visually on the screen, an algorithm that surpasses the ability of human inspection is necessary.

Another interpretation of “segmentation” refers to Piecewise Linear Approximation (PLA). The goal here is to approximate a time series ***T*** with a more compact representation by fitting *k* piecewise polynomials using linear interpolation or linear regression, while minimizing the error with respect to the original ***T*** (Harguess and Aggarwal 2009; Wang et al. 2011). Success here is measured in terms of root-mean-squared-error, and it does not (in general) indicate any *semantic* meaning of the solution.

Finally, we are not interested in segmenting individual phrases/gestures/phonemes etc. This type of work is almost always heavily domain dependent and requires substantial training data (Aoki et al. 2016). For example, here is a significant amount of work that attempts to segment the time series equivalent of the string *nowthatchersdead* to produce “now *thatchers* dead” (and not “now that *chers* dead”). In contrast, we are interested in segmenting at a higher level, which would be the equivalent of segmenting an entire book into chapters or themes.

### Related work

Hidden Markov Models (HMMs) have been successfully used to segment discrete strings. Examples of this include segmenting a DNA strand into coding and non-coding regions, and there are efforts to use HMMs in the real-valued space (but they are almost always tied to a single domain, such as seismology (Cassisi et al. 2016)). We have considered and dismissed HMMs for several reasons. To use HMMs with real-valued time series, we must set *at least* two parameters, the level of cardinality reduction (the number of states to discretize to) and the level of dimensionality reduction (the number of values to average) (Cassisi et al. 2016). This is in addition to specifying the HMM architecture, which is tricky even for domain experts (Cassisi et al. 2016) and contrary to our hope for a domain agnostic algorithm.

The work that most closely aligns with our goals is Autoplait (Matsubara et al. 2014a), which segments time series using Minimum Description Length (MDL) to score alterative HMMs of the data. This work also stresses the need for domain independence and few parameters. The most significant limitation of Autoplait is that it is only defined for the batch case. It would not be trivial to convert it to handle streaming data. This approach requires discrete data, which is obtained by an equal division of the range bound by the smallest and largest values seen. In the streaming case, wandering baseline or linear drift ensures that at some point all the incoming values are greater (or smaller) than the values the model can process. This is surely not unfixable, but it is also not simple to address, and it is only one of the many issues that must be overcome to allow an Autoplait variant to handle streaming data.

The authors of Autoplait (and various subsets thereof) have many additional papers in this general space. However, to the best of our understanding, none of them offer a solution for the task-at-hand. For example, while StreamScan is a streaming algorithm (Matsubara et al. 2014b), the authors note the need to train it: “*we trained several basic motions, such as* ‘*walking,’* ‘*jumping’*” (our emphasis), and the algorithm has at least six parameters.

## Semantic segmentation

We are now able to formally define the task-at-hand. Assume we have a system ***S***, which can be in two or more discrete states (or *regimes*). Examples of such systems include:

- The heart of a patient recovering from open heart surgery. The patient’s heart may be in the state of *tamponade* or *normal* (Chuttani et al. 1994).
- A music performance may often be envisioned a system that moves between the states of *intro*, *verse*, *chorus*, *bridge*, and *outro* (Serra et al. 2014).
- Fractional distillation of petrochemicals contains cycles of *heating*, *vaporizing*, *condensing*, and *collecting* (Nishino et al. 2003).
- An exercise routine often consists of *warm*-*up*, *stretching*, *resistance training*, and *cool*-*down*. This special case of treating human behavior as a switching linear dynamic system (SLDS) (Pavlovic et al. 2001) has become an increasingly popular tool for modeling human dynamics (Bregler 1997; Reiss and Stricker 2012).

We can monitor most of these systems with sensors. For the cases mentioned above, a photoplethysmograph, a microphone, a thermocouple, and a wrist-mounted accelerometer (smartwatch) are obvious choices. In most cases, one would expect the time series from the sensors to reflect the current state of the underlying system. This understanding allows us to produce the following definition of the problem regarding the time series semantic segmentation task:

### **Definition 4**

Given a time series ***T***, monitoring some aspect of a system ***S***, infer the boundaries ***b*** between changes of state.

We recognize that this definition makes some simplifying assumptions. Some systems are not naturally in discrete states, but may be best modelled as having a degree of membership to various states. For example, *Hypokalemia*, a disease where the heart system is deficient in potassium, is often diagnosed by examining ECGS for increased amplitude and width of the P-wave (Weiner and Charles 1997). Hypokalemia can manifest itself continuously at any level from mild to severe. In fact, our example of tamponade is one of the few intrinsically discrete heart conditions. Nevertheless, many systems *do* switch between discrete classes, and these are our domains of interest. Even though hypokalemia can *change* continuously, in practice it often changes fast enough (in response to intravenous or oral potassium supplements) to be detectible as a regimen change in a window of ten minutes, and we can easily support windows of this length or greater.

Note that even in systems that do have some mechanism to “snap” the system to discrete behaviors, there is often another ill-defined “other” class. For example, consider the short section of time series shown in Fig. 1.

Here the need for precise movements forces the exercise repetitions to be highly conserved. However, there is no reason to expect the transitions between the repetition sets to be conserved.

Similar remarks apply to many other domains. In many cases, the majority of the data examined may consist of ill-defined and high entropy regions. Note that these observations cannot be used to conclude that the underlying system is not in any state. It may simply be the case that the view given by our sensor is not adequate to make this a determination. For example, a sensor on the ankle will help distinguish between the states of walking and running, but it will presumably offer little information when the system (the human) is toggling between typing and mouse-use.

### Introduction FLUSS

We begin by introducing FLUSS (Fast Low-cost Unipotent Semantic Segmentation), an algorithm that extends and modifies the (unnamed) algorithm hinted at (Yeh et al. 2016). Later, in Sect. 3.3, we will show how to take this intrinsically batch algorithm and make it a streaming algorithm. For clarity of presentation we begin by only considering the single dimensional case and show the trivial steps to generalize to the multi-dimensional case in Sect. 3.4.

The task of FLUSS is to produce a companion time series called the Arc Curve (AC), which annotates the raw time series with information about the likelihood of a regime change at each location. We also need to provide an algorithm to examine this Arc Curve and decide how many (if any) regimes exist.; that issue is considered separately in Sect. 3.2.

**Fig. 2:**
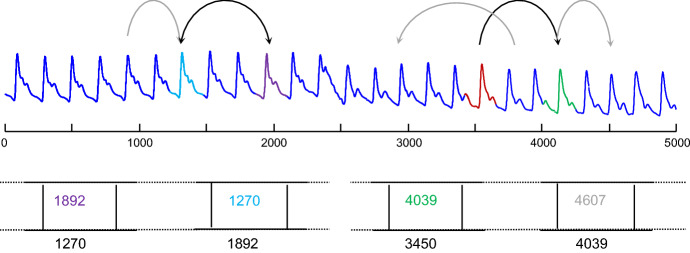
Selected arcs illustrated with the corresponding Matrix Profile indices indicated. Note that nearest neighbor subsequences indices can be symmetric, e.g., 1270 and 1892, but this is not generally true. A subsequence’s nearest neighbors can be located to the left or to the right

FLUSS takes both a time series ***T*** and a user provided subsequence length as inputs, and outputs an AC vector of length *n*, where each index *i* contains the number of “arcs” that cross over *i*. We define an “arc” as follows: the *i*^*th*^ entry in the *MPIndex* vector contains a positive integer *j*, which indicates the nearest neighbor location. So, for the *i*^*th*^ entry, containing a positive integer *j*, the nearest neighbor for the time series subsequence beginning at index *i* is the time series subsequence beginning at index *j*. We can visualize each entry pair (*i*,*j*) as an arc drawn from location *i* to *j*. The spatial layout of the arcs, along with the number of “arc” crossing over of each index *i,* is summarized by the Arc Curve. Specifically, index *i* of the Arc Curve contains a non-negative integer indicating the number of arcs that cross over *i*. Figure 2 below illustrates this notation.

Note that every index has exactly one arc leaving it; however, each index may have zero, one, or multiple arcs pointing to it. We define the Arc Curve more formally below:

#### **Definition 5**

The Arc Curve (AC) for a time series ***T*** of length *n* is itself a time series of also length *n* containing non-negative integer values. The *i*^*th*^ index in the AC specifies how many nearest neighbor arcs from the *MPIndex* spatially cross over location *i*.

Now, we can state the intuition of our segmentation algorithms.

*Our Overarching Intuition* Suppose a time series ***T*** has a regime change at location *i*. We would expect few arcs to cross *i*, as most subsequences will find their nearest neighbor *within* their host regime. Thus, the height of the Arc Curve should be the lowest at the location of the boundary between the change of regimes/states.

**Fig. 3:**
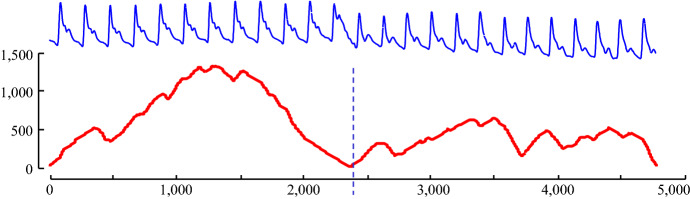
(Top) The ABP of a reclining male. At time 2400 he was rotated into a standing position. (bottom) The AC plot for this dataset shows a clear valley at time of system change

Figure 3, shows the AC plot for the dataset shown in Fig. 2, which will be used as a running example. We consider the Arterial Blood Pressure (ABP) of a healthy volunteer resting on a medical tilt table (Heldt et al. 2003). At time 2400, the table was tilted upright, invoking a response from the homeostatic reflex mechanism.

While the figure above hints at the utility of FLUSS, it also highlights a weakness. Note that while the Arc Curve has a satisfyingly low value at the location of the regime change, it also has low values at both the leftmost and rightmost edges. This occurs because there are fewer candidate arcs that can cross a given location at the edges. We need to compensate for this bias, or false positives are likely to be reported near the edges.

**Fig. 4:**
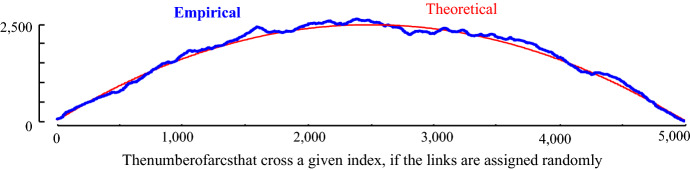
The Idealized Arc Curve (IAC) for a time series with no localized similarity structure is an inverted parabola with a height ½*n*. An empirical curve shows close agreement. As we will see later, it is actually a special case of beta (2, 2, a, c)

This compensation is easy to achieve. We begin by imagining the case where there is no locality structure in the time series under consideration; for example, imagine we are examining a random vector. Under such circumstances, we would expect the arcs from each subsequence to point to an effectively random location. Given this null case, with no structure, what would an Idealized Arc Curve (IAC) look like? With a little introspection, one can see that, as shown in Fig. 4, it would be an inverted parabola with its height ½*n* (we relegate the derivation of this fact to (Keogh 2017)).

To compensate for the edge effect bias in the Arc Curve, for each location *i*, we consider the actual number of observed arc crossings relative to the number of expected arc crossings predicted by our parabolic model (1), to obtain the Corrected Arc Crossings (CAC):

1 $$ CAC_{i} = min\left( {\frac{{AC_{i} }}{{IAC_{i} }}, 1} \right) $$

The *min* function is to keep the CAC bounded between 0 and 1 in the logically possible (but never empirically observed) case that AC_*i*_ > IAC_*i*_.

This normalized and bounded measure is useful because it allows the following:

- Commensurate comparisons across streams monitored at different sampling rates.
- The possibility to learn domain specific *threshold* values. For example, suppose we learn in ECG training data, that for patient in an ICU recovering from heart surgery, a CAC value less than 0.2 is rarely seen unless a patient has cardiac tamponade. Now we can monitor and alert for this condition.

**Fig. 5:**
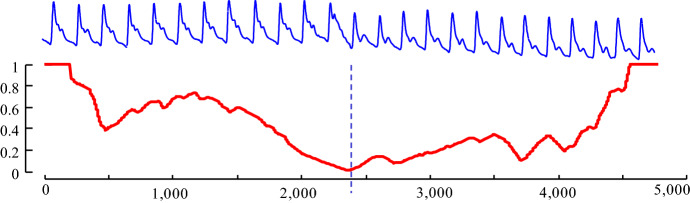
(Contrast with Fig. 3). (top) Our running ABP example. (bottom) The CAC minimizes in the correct place and avoids the “edge-effect” false positives of the AC curve

Figure 5, shows the CAC for our running example. Note that the issue of the edge bias of AC has been resolved, and the curve minimizes at the correct location of 2400.

Before continuing, we will demonstrate the invariance of the CAC to several issues commonly encountered during real-world uses. The CAC for our running example was recomputed after modifying the original data in several ways, including:

- Downsampling from the original 250 Hz to 125 Hz (red).
- Reducing the bit depth from 64-bit to 8-bit (blue).
- Adding a linear trend of ten degrees (cyan).
- Adding twenty dB of white noise (black).
- Smoothing, with MATLAB’s default settings (pink).
- Randomly deleting 3% of the data, and filling it back in with simple linear interpolation (green).

**Fig. 6:**
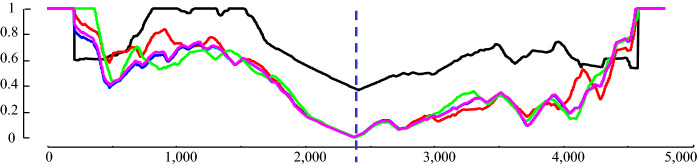
The CAC computed for our running example after it was distorted in various ways (best viewed in color; key is above). Contrast with Fig. 5. Note that the cyan line is hard to see, as it mostly falls beneath the pink line (Color figure online)

As Fig. 6 suggests (at least for this example), the CAC is quite robust regarding these issues, and the minimum value of the CAC still occurs in the same place.

**Fig. 7:**
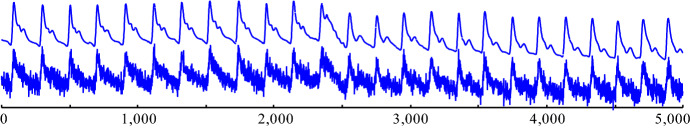
A comparison of the original TiltABP (top) and the data with twenty dB of noise added (bottom)

The only distortion to appreciably change the CAC is noise; however, as Fig. 7 shows, we added an enormous amount of noise, and we still found the correct segmentation.

We have shown that the CAC is robust to many variations of time series data, and are now ready to fully explain the algorithm for obtaining the CAC. While the construction of the CAC is straightforward, given the discussion above, we formalize it on Table 1 for clarity.

**Table 1:**
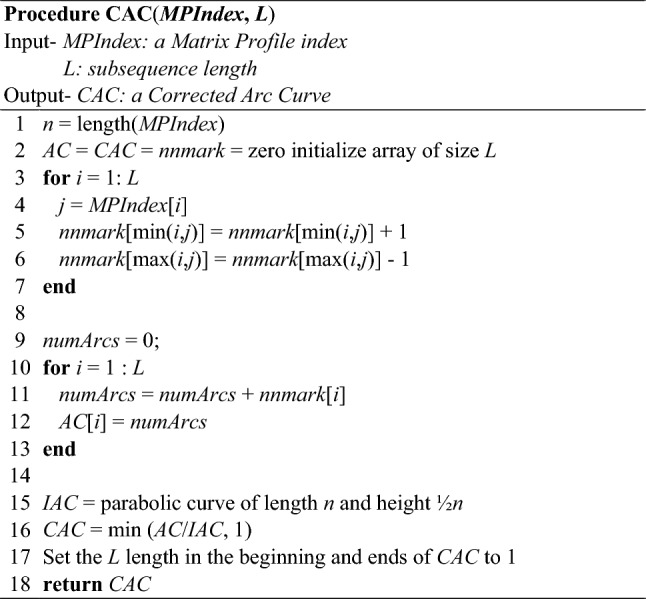
Algorithm for construction CAC

In lines 1–2, we obtain the length of the *MPIndex* and zero initialize three vectors. Next, we iterate over the *MPIndex* to count the number of arcs that cross over index *i* in lines 3 through 7. This information is stored in *nnmark*. Then, we iterate over *nnmark* and cumulatively sum its values consecutively for each index *i*. The cumulative sum at *i* is stored in AC*i*. This is accomplished in lines 10–13. Finally, in lines 15–18, we normalize AC with the corresponding parabolic curve to obtain the CAC.

### Extracting regimes from the CAC

With our CAC defined, we are now ready to explain how to extract the locations of the regime changes from the CAC. Our basic regime extracting algorithm requires the user to input *k*, the number of regimes. This is similar to many popular clustering algorithms, such as *k*-means, which require the user to input the *k* number of clusters. Later we will demonstrate a technique to remove the need to specify *k,* given some training data to learn from (see Sect. 4.3).

We assume here that the regimes are distinct, for example walk, jog, run. If a regime can be repeated, say walk, jog, walk, our algorithm may have difficulties; that issue will be dealt with in Sect. 3.5.

As hinted in Fig. 5, a small value for the lowest “valley” at location *x* is robust evidence of a regime change at that location. This is based on the intuition that a significantly fewer number of arcs would cross location *x* if *x* is a boundary point between two discrete states (Yeh et al. 2016). Note that this intuition is somewhat asymmetric. A large value for the lowest valley indicates that there is no evidence of a regime change, not that there is positive evidence of no regime change. This is a subtle distinction, but it is worth stating explicitly.

At a high level, the Regime Extracting Algorithm (REA) searches for *k* lowest “valley” points in the CAC. However, one needs to avoid the trivial minimum; if *x* is the lowest point, then it is almost certain that either *x *+ 1 or *x*−1 is the second lowest point. To avoid this, FLUSS does not simply return the *k* minimum values. Instead, it obtains one minimum “valley” value at location *x*. Then, FLUSS sets up an exclusion zone surrounding *x*. For simplicity, we have defined the zone as five times the subsequence length both before and after *x*. This exclusion zone is based on an assumption the segmentation algorithm makes, which is that patterns must have multiple repetitions; FLUSS is not able to segment *single* gesture patterns. With the first exclusion zone in place, FLUSS repeats the process described above until all *k* boundary points are found.

While this algorithm is obvious and intuitive, for concreteness, we formally outline in Table 2. Note that *numRegimes* is not a parameter of the segmentation algorithm per se; it is a parameter of our regime extraction algorithm. By analogy, you can build a dendrogram without specifying parameters, but any algorithm to convert it to partitional clusters will always have a parameter. There may be other ways to extract regime; Table 2 just offers a concrete and simple method.

**Table 2:**
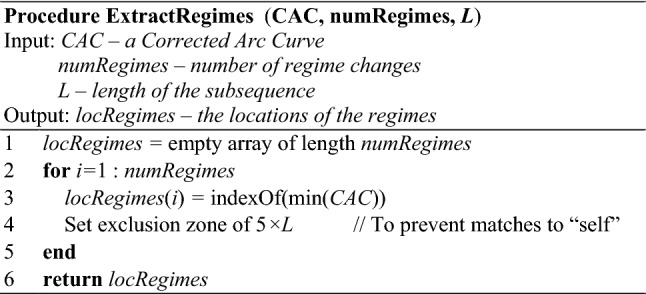
REA: Algorithm for extracting regimes

### Introducing FLOSS

In the previous sections, we have shown that at least in our running example, FLUSS can detect changes of regimes in batch datasets. We now turn our attention the streaming case, in which we maintain the CAC over a sliding window; an example of this could be the last ten minutes of a patient recovering from heart surgery. In principle, this seems simple. At every time stamp, we need to ingress the newly arriving point, and egress the oldest point, updating all the arcs in the Matrix Profile index and adjusting the CAC as needed. However, there is a large asymmetry in the time complexity for ingress and egress.

- *Ingress* When the new point arrives, we must find its nearest neighbor in the sliding window, and determine whether any item currently in the sliding window needs to change its nearest neighbor to the newly arrived subsequence. Using the MASS algorithm, this takes just O(*n*log*n*) (Mueen et al. 2015).
- *Egress* When a point is ejected, we must update all subsequences in the sliding window that currently point to that departing subsequence (if any). This is a problem, because while pathological unlikely, almost *all* subsequences could point to the disappearing subsequence. This would force us to do O(*n*^2^) work, forcing us to re-compute the Matrix Profile (Yeh et al. 2016).

This issue would not exist if the arcs in the Matrix Profile only went in one direction, to a *previous* time. In that case, when we egress a data point, for the corresponding subsequence being removed:

- As the arcs only go to a previous time, we do not have to delete arcs that point to it, since it does not have one.
- As for the arcs that point away from it, we could delete that arc by removing the first element in the Matrix Profile index in O(1).

This would indicate that the overall time to maintain the 1-Direction on CAC O(*n*log*n*) for ingress plus O(1) for egress, for a total of O(*n*log*n*).

**Fig. 8:**
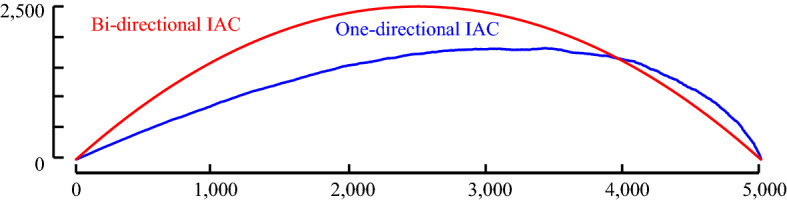
The one-directional IAC (IAC_1D_) is a right skewed distribution with shorter height than the original IAC

However, this begs the question, would using the CAC_1D_ yield similar results to using the CAC? To test this, we begin by computing the empirical one-directional IAC (IAC_1D_). The empirical IAC_1D_ is shown with the theoretical original (bi-directional) IAC in Fig. 8.

**Fig. 9:**
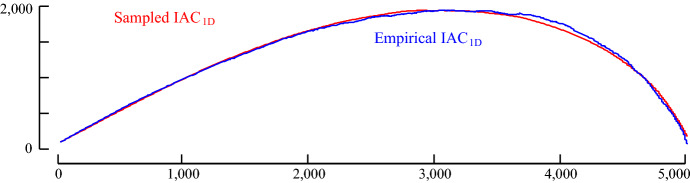
The empirical IAC_1D_ is modeled closely by a beta distribution

Compared to the original IAC, IAC1D has a somewhat similar shape, but it is shorter and skewed to the right. The skewness is caused by the fact that it is more likely for arcs to cross later in time, since all the arcs are pointing forward in time. By theoretical modeling/visual inspection (Keogh 2017), we note that the distribution of IAC_1D_ can be modeled by a beta distribution. The empirical IAC_1D_ and a sample generated by the beta distribution is shown in Fig. 9. Note that in retrospect, we can see the parabolic curve of Fig. 4 was just a special case of the beta distribution with α = β = 2.

**Fig. 10:**
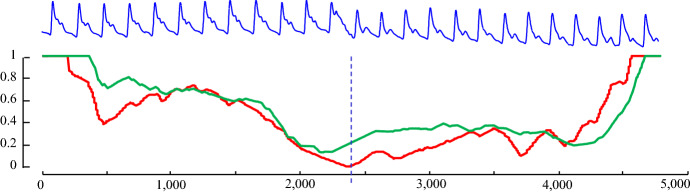
(Top) Our running ABP example. *bottom*) the CAC (red) from Fig. 5 and the CAC_1D_ (green) (Color figure online)

As a result of this difference, IAC_1D_ is used instead of IAC when computing CAC_1D_. We then, computed the CAC_1D_ on our running example, which is shown in Fig. 10.

### Generalizing to multi-dimensional time series

For some applications, single dimensional data may not be sufficient to distinguish between the regimes. In such cases, one may benefit from considering additional dimensions. Below we show an intuitive motivating toy example of this, before discussing the trivial changes in our framework to leverage additional dimensions.

Consider the classic CMU Mo-Cap dataset (Mocap.cs.cmu.edu 2017). Among the activities in this archive, we choose three sample activities to demonstrate our point: basketball-forward-dribble, walk-with-wild-legs and normal-walk. Intuitively, we might expect that using *just* sensor data from the hand or foot data is sub-optimal for this segmentation task. For example, while the hand data can differentiate basketball-forward-dribbling from either normal-walk or walk-with-wild-legs, it cannot be used to differentiate normal-walk from walk-with-wild-legs. In this case, data is needed from another source such as foot, which can be seen as an “expert” in gait activities.

**Fig. 11:**
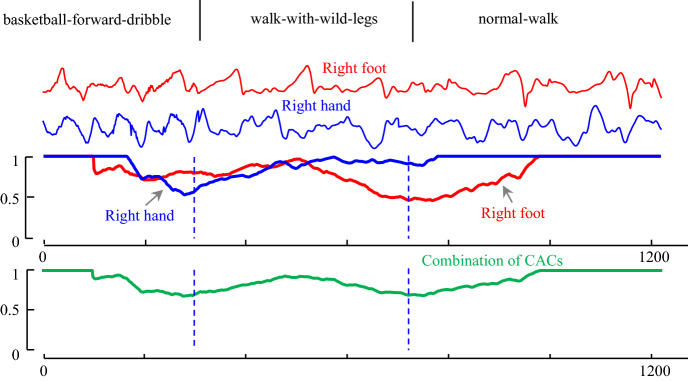
An example of multi-dimensional time series segmentation on three activities: basketball-forward-dribble, walk-with-wild-legs and normal-walk from CMU dataset. (top) The CAC obtained from using right hand data (red) and right foot data (blue) which each of them separately cannot be used for segmentation. (bottom) the combination of two CACs which can segment all three activities (Color figure online)

However, we might imagine that by using *both* data sources, all three activities can be mutually distinguished. This only leaves the question of how best to combine information from multiple time series. As Fig. 11 suggests, this is actually very easy with our framework, one can simply take the mean of two or more CAC’s to produce a single CAC that pools information from multiple sources.

While Fig. 11 visually hints at the utility of combining dimensions, one can also *objectively* measure the improvement. Our formal discussion of such an objective score is in Sect. 3.6, but previewing it, we have a segmentation scoring function that gives zero for a segmentation, which exactly agrees with the ground truth. The score of segmentation by using just the foot or just the hand data are 0.27, 0.28 respectively. The score of using both is dramatically improved to just 0.05.

Note that this method of combining information from different sensors in the CAC space has a very useful and desirable property. Because each CAC is already normalized to be between zero and one, it does not matter if the sensors are recording the data at different sampling rates or precisions. For example, in Fig. 11 the data was recorded at 120 Hz for both right foot and right hand. However, if we downsample, to just the right hand to 40 Hz, the resulting combinations of CACs is visually indistinguishable from the one shown (in green) in Fig. 11.*botttom*.

### Adding a temporal constraint

**Fig. 12:**
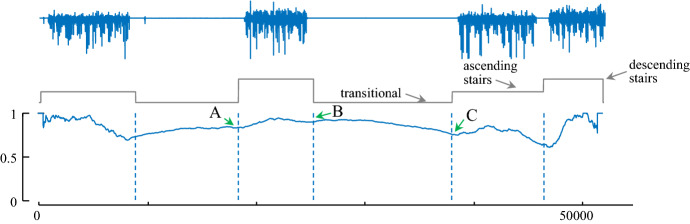
(Top to bottom) The accelerometer’s X data of shoe from PAMAP Dataset-Subject1 (blue), its ground truth segmentation, into ascending stairs, descending stairs and transitional activities (gray) which includes some low amplitude noise data. The classic CAC produces false negatives at locations A, B and C (Color figure online)

There are some situations in which CAC may have difficulty in detecting a change of regime. Consider Fig. 12.*top*, taken from the PAMAP dataset (Reiss and Stricker 2012), which shows an accelerometer trace of an individual walking up, then down a flight of stairs *twice*, with long “transition” rest periods in-between. Note that these transitional periods look constant at the plotted scale but contain low amplitude random movements as the individual rests.

While the CAC correctly detects *some* transitions, there are three obvious false negatives in locations denoted A, B and C. The reason for these false negatives is existence of multiple periods of the same regime, which are similar but disconnected. For example, there is a region of ascending stairs, followed by a transistion period, then descending stairs, and *another* period of ascending stairs. One might expect that approximately half the arcs that originate in the first section of ascending stairs (and vice versa), will point to the second section, crossing over the two transitions in-between, and robbing us of the arc “vacuum” clue the CAC exploits (recall Fig. 2).

This issue can occur in multiple domains. For example, after heart surgery some patients may exhibit occasional symptoms of *Pulsus Paradoxus* (Chuttani et al. 1994), as they adopt different sleeping postures (i.e. rolling onto their sides). The experiments in Sect. 4 suggest that if the CAC is computed on say, any one-minute snippet of PPG time series, it can robustly detect transitions between normal heartbeats and Pulsus (if present). However, while segmenting hour-long snippets is computationally trivial, many of the arcs between healthy heartbeats will span tens of minutes, and cross over the (typically) shorter regions of Pulsus, effectively “blurring” the expected decrease in the number of arcs that signals a change of boundaries.

We can solve this problem by adding a *Temporal Constraint TC*. In essence, even if examining a long or unbounded time series, the algorithm is constrained to only consider a local temporal region when computing the CAC. Note that this constraint does not increase the computational time; in fact, it reduces that. We can create such a constraint easily if we simply ensure that the arcs cannot point to subsequences further away than a user-specified distance. In this solution, we just need to set one parameter, *TC*, which corresponds to the approximate *maximum* length of segment in our domain. For example, there has been a lot of interest in segmenting repetitive exercise (Morris et al. 2014) using wearables. While the length of time for a ‘set’ depends on the individual and the apparatus (i.e. dumbbell vs. barbell), virtually all sets last no more than 30 s (Morris et al. 2014), thus we can set *TC *= 30. For intuitiveness, we discuss *TC* in wall-clock time; however, internally we convert it to some integer based on the sampling rate. For example, for the PAMAP dataset which is sampled at 100 Hz, a *TC* of 30 restricts the length of all arcs to less than 3000 = 100 × 30.

**Fig. 13:**
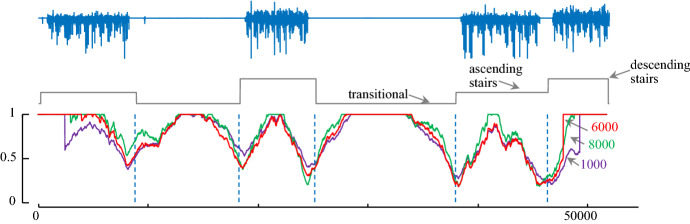
(Top to bottom) The accelerometer’s X data of shoe from PAMAP Dataset-Subject1 (blue), its ground truth segmentation, into ascending stairs, descending stairs and transitional activities (gray) which includes some noise data. CAC after applying Arc Constraint with the length of constraint 6000, 8000 and 1000. Contrast with Fig. 12 (Color figure online)

To test the utility of temporal constraints, we revisit the PAMAP dataset (Reiss and Stricker 2012) snippet shown in Fig. 12.*top*. As shown in Fig. 13, even for a wide range of values for the temporal constraint, the CAC can detect the regime changes accurately. Note that because this dataset is only “weakly labeled”, the CAC result is not precisely aligned with boundaries given by the original authors, but it is subjectively correct by visual inspection.

Recall that we correct the IAC to the CAC based on the assumption that in a time series with no locality structure, the arcs from each subsequence point to an effectively random location. However, when using the temporal constraint, the arcs cannot point to any arbitrary location. Thus, the previous assumption is no longer useful here. Nonetheless, here the correction “curve”, instead of being parabolic or a beta distribution, is simply a uniform distribution, except for the first *TC *×* sampling*-*rate,* and last *TC *×* sampling*-*rate* data points. As these are asymptotically irrelevant, as shown in Fig. 13, we simply hardcode the corresponding CAC to one in these regions. Note that temporal constraints require you to make some assumptions about the domain in question. This experiment suggests that if your assumptions are reasonable, this algorithm will work well. If your assumptions are strongly violated, we make no claims.

### Scoring function

Most of the evaluations of segmentation algorithms have been largely anecdotal (see (Lin et al. 2016) for a detailed survey), and indeed we also show visually convincing examples in Sect. 4. Because of the scale of our experiments, however, as thirty-two diverse datasets are examined, we need to have a principled scoring metric.

Many research efforts have used the familiar precision/recall or measures derived from them. However, as (Lin et al. 2016) points out, this presents a problem. Suppose the ground truth for transition between two semantic regimes is at location 10,700. If an algorithm predicts the location of the transition at say 10,701, should we score this as a success? What about, say, 10,759? To mitigate this brittleness, several authors have independently suggested a “Temporal Tolerance” parameter to bracket the ground truth (Lin et al. 2016). Yet, this only slightly mitigates the issue. Suppose we bracket our running example with a range of 100, and reward any prediction in the range 10,700 ± 100. Would we penalize an algorithm that predicted 10,801, but reward an algorithm that predicted 10,800?

Another issue in creating a scoring function is rewarding a solution that has *k* boundaries predictions, in which most of the predictions are good, but just one (or a few) is poor. If we insist on a one-to-one mapping of the predictions with the ground truth, we over-penalize any solution for missing one boundary while accurately detecting others (a similar matching issue is understood in many biometric matching algorithms).

**Fig. 14:**
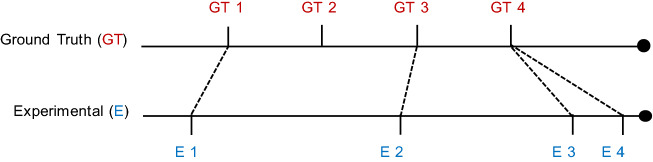
An example of our scoring function in action. The top line illustrates the locations of the ground truth locations (GT1, GT2, GT3, GT4), and the bottom line illustrates the boundary locations (E1, E2, E3, E4) reported by an algorithm. Note that multiple proposed boundary points may be mapped to a single ground truth point

The solution is visually explained in Fig. 14, and formally outlined in Table 3. It gives 0 as the best score and 1 as the worst. The function sums the distances between the ground truth boundary points and the boundary points suggested by an algorithm; that sum is divided by the product of the number of segments, and then the length of the time series to normalize the range to [0, 1].

**Table 3:**
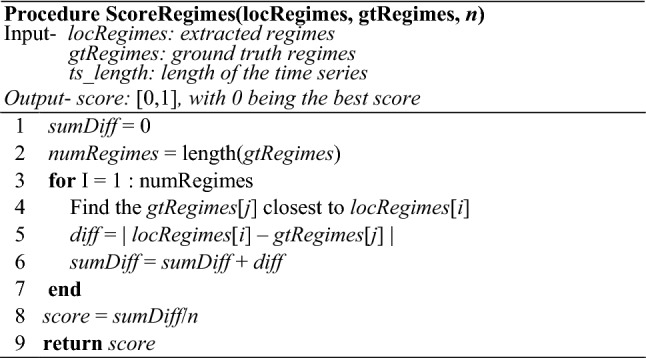
Scoring function algorithm

## Experimental evaluation

We begin by stating our experimental philosophy. We have designed all experiments such that they are easily reproducible. To this end, we have built a Web page (Keogh 2017) that contains all of the datasets and code used in this work as well as the spreadsheets containing the raw numbers and some supporting videos. The thirty-two benchmark segmentation test datasets we created, in addition to the case study datasets, will be archived in perpetuity at (Keogh 2017), independent of this work. We hope the archive will grow as the community donates additional datasets.

### Benchmark datasets

We created an extremely diverse collection of benchmark test datasets. The biological datasets include time series taken from humans, birds, rats, pigs, and insects. The mechanical datasets include data taken from robots and electrical power demand (both from a single building and an entire city). The datasets fall into three categories:

- *Synthetic* There is one completely synthetic dataset, mostly for calibration and sanity checks.
- *Real* The majority of our datasets are real. In most cases, the ground truth boundaries are confidently known because of *external* information. For example, for the *Pulsus Paradoxus* datasets (Chuttani et al. 1994), the boundaries were determined by the attending physician viewing the patient’s Echocardiogram.
- *Semi-Real* In some cases, we contrived real data to have boundaries. For example, we took calls from a single species of bird that were recorded at different locations (thus they were almost certainly different *individuals*) and concatenated them. Thus, we expect the change of *individual* to also be a change of *regime*.

**Fig. 15:**
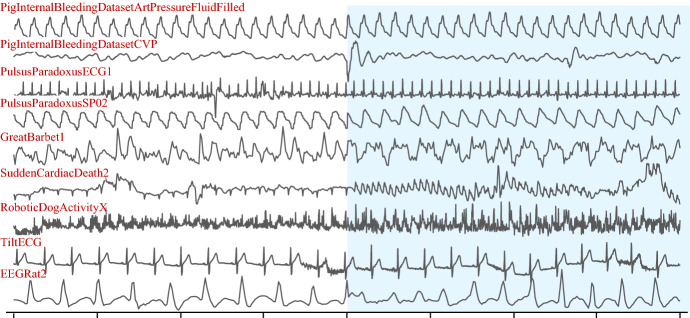
Snippets (not the full traces) from a random selection of the test datasets. The snippets are centered on a boundary (change of background color). There are no axes in this figure, as the data are at different scales

As Fig. 15 suggests, some of the boundaries are obvious visually. However, we can also see that many are so subtle that finding the boundary is a non-trivial challenge for humans, including domain experts (in fairness, these are only snippets, the excluded data would probably give the human more context).

For brevity, we omit further discussion of these datasets. However, we have created a visual key, which gives detailed information on the provenance of each dataset and placed it in perpetuity at (Keogh 2017).

For these experiments, we set the only parameter, the subsequence length *L*, by a one-time quick visual inspection. We set it to be about one period length (i.e. one heartbeat, one gait cycle, etc.). As we will show in Sect. 4.6, our algorithm is not sensitive to this choice. However, as we will show in several of our case studies, it is typically very easy to learn this parameter directly from the data, even if only one regime is available for the parameter learning algorithm.

### Rival methods

The most obvious rival method is Autoplait (Matsubara et al. 2014a). As noted above, while there are dozens of segmentation algorithms, it is often difficult or unfair to compare to them because:

- They are designed only for a limited domain; thus, if they are not competitive, it might be because they are just not suited for some or most of the diverse datasets considered.
- They require the setting of many parameters; if they are not competitive, it might be because we tuned parameters poorly.
- The code is not publicly available; if they are not competitive, it might be because of our unconscious *implementation bias* (Keogh and Kasetty 2003).

In contrast to all of the above, Autoplait is domain agnostic, parameter-free, and the authors make their high-quality implementation freely available (Matsubara et al. 2014a) and are even kind enough to answer questions about the algorithm/code.

Autoplait segments time series by using MDL to recursively test if a region is best modeled by one HMM or two (this is a simplification of this innovative work, we encourage the interested reader to refer to the original paper (Matsubara et al. 2014a)).

After confirming that we had the code working correctly by testing over the authors’ own datasets and some toy datasets, we found that Autoplait only produced a segmentation on 12 out of our 32 test datasets. The underlying MDL model seems to be too conservative. To fix this issue, for every dataset we carefully hand-tuned a parameter W, which we used to reduce the weight of their Cost(***T***|***M***), making the splits “cheaper,” encouraging the production of *k* regimes. This is the only change we made to the Autoplait code. With this change, most, but not all, datasets produced a segmentation. We found that we could perfectly replicate the results in the original Autoplait paper, on the authors own chosen benchmark datasets. However, because these datasets are not very challenging, we confine these results to our supporting webpage (Keogh 2017).

We also compared it to the HOG_1D_ algorithm (Zhao and Itti 2016), which has similar goals/motivations to FLOSS, but is batch only.

### Case study: hemodynamics

In this case study, we revisit our running example in more depth. Recall that in Sect. 3.1 we suggested that in some domains it may be possible to use training data to learn a value for the CAC score that indicates a change of regime, and we expect that value to generalize to unseen data from the same domain. To test this notion, we consider the Physiologic Response to Changes in Posture (PRCP) dataset (Heldt et al. 2003).

**Fig. 16:**
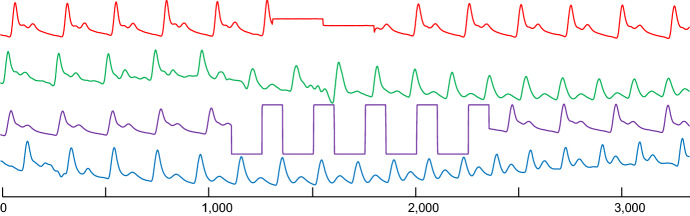
Four random examples of “no-regime-change.” Even these regions of “no change” include sensor artifacts, wandering baseline, noise, and short disconnection artifacts

The PRCP dataset consists of continuously monitored Arterial Blood Pressure (ABP) of ten healthy volunteers (five of each sex). During sessions lasting approximately one hour each, the subject’s posture was changed in two ways; by rotating the medical tilt table they rested on, or by asking the subject to arise from the reclined table under their own power. Each of these posture-change events (just ‘events’ in the below) was separated by five minutes in the resting supine position. Because the timing of these events was carefully recorded, this dataset offers an objective ground truth for regime change. Note that our running example showed a “clean” snippet from this domain for clarity, but as Fig. 16 shows, this data can be more complex.

To avoid cherry picking, we chose the first subject (in the original archive), a male, to use as the training data. Likewise, to avoid parameter tuning, we googled “normal resting bpm.” The first result, from the Mayo Clinic, suggested “60–100 bpm”, so we set the subsequence length to 187, which at 250 Hz corresponds to the average of these values.

As we are attempting to learn from only negative examples, we manually selected twenty regions, each one-minute long (possibly with overlaps) from the regions that do not include any event. For our testing data, we selected 140 negative and 60 positive (regions that straddle an event) from the remaining nine traces.

**Fig. 17:**
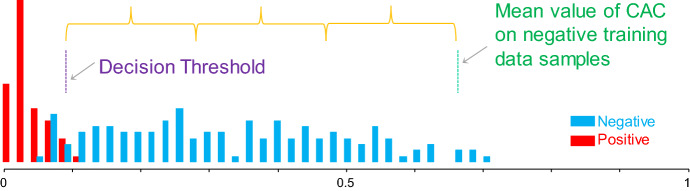
The positive and negative holdout data can be classified by a simple decision threshold; the mean of the training negative examples minus three standard deviations

We ran FLUSS on the twenty training objects that recorded the minimum CAC value encountered. As shown in Fig. 17 (left), the mean value was 0.671 with a standard deviation 0.194. Using the classic statistical-process-control heuristic, we set the threshold for the testing phase to the mean minus three standard deviations, or 0.089. As we can see in Fig. 17 (right), this gives us an accuracy of 93.5%, with one false negative and twelve false positives.

Note that we cannot guarantee here that the false positives are really “false”. Independent of the externally imposed interventions, the subject may have induced a regime change by thinking about a stressful situation (Maschke and Scalabrini 2005). Further note that we could have improved these results significantly with a little work. For example, we could have tuned *L*, the only parameter, we could have built a separate model for females, or for overweight individuals, we could have removed noise or wandering baseline (a common practice for such data) etc. Nevertheless, this experiment bodes well for our claim that we can learn a domain dependent threshold for flagging regime changes, and then it will generalize to unseen data.

### User study: comparisons to human performance

As noted above, the evaluation of semantic segmentation algorithms has often been anecdotal and visual (Lin et al. 2016). In essence, many researchers overlay the results of the segmentation on the original data, and we invite the reader to confirm that it matches human intuition (Bouchard and Badler 2007; Matsubara et al. 2014a; Lin et al. 2016; Aminikhanghahi and Cook 2017). While we are not discounting the utility of such sanity checks (see Fig. 23), by definition, such demonstrations can only offer evidence that the system is par-human (Anonymous 2018). It is natural to wonder if semantic segmentation can achieve performance at human levels. To test this, we performed a small user-study. We asked graduate students in a data mining class to participate. Participation was voluntary and anonymous; however, to ensure that the participants were motivated to give their best effort, a cash prize was given for the best performance.

The study was conducted as follows. The participants were briefed on the purpose and meaning of semantic segmentation and where shown some simple annotated examples (This briefing is archived in (Keogh 2017)). Then, they were given access to an interface that showed twelve random examples^1^ in a serial fashion from the archive discussed in Sect. 4.1. The interface allowed the participants to explore the data at their leisure and then click on the screen to denote their best guess as to the location of the regime change.

Because our scoring function is fine-grained, we only count a method as *wining* if its score is less than half the score of its rival. Otherwise, we report a *tie*. Table 4 summarizes the outcomes.

**Table 4:** The performance of fluss versus humans

	FLUSS	Best human	Ave human
Mean score	0.013	0.011	0.120
Win | lose | draw over FLUSS	NA	2 | 4 | 6	0.81 | 9.5 | 2.0

While the scale of this experiment was modest, these results suggest that we are at, or are approaching, human performance for semantic segmentation of time series.

### Comparisons to rival methods

Despite our best efforts, we could not get the original Autoplait algorithm to produce any segmentation on 20 of our 32 test datasets. We counted this as a “loss” for Autoplait “classic”. By carefully adapting the algorithm (see Sect. 4.2) we could get Autoplait to produce a segmentation on thirteen additional datasets “Autoplait Adapted”. On the datasets it did predict segmentations for, it sometimes predicted too many or too few segments. In those cases, we allowed both versions to “cheat”. If it predicted too few segments, we took only the closest matches, and gave it all the missing matches with no penalty. If it predicted too many segments, we only considered the best interpretation of a subset of its results without penalizing the spurious segments.

In contrast, HOG_1D_ only refused to produce a segmentation on 2 of our 32 datasets. For the rest, it was able to produce the required *k* splits.

Recall that we tested twenty-two humans on a subset of the data. We invited the best scoring individual to segment all the data, motivated to produce his best effort by a financial incentive. Finally, for calibration, similarly to the default-rate of classification, we considered a random algorithm, which was allowed 100 attempts at guessing the segmentation and reports the average of all attempts. As we noted above, we set the only parameter, the subsequence length *L*, to be about one period length before we did any experiments. The result is shown in Table 5.

**Table 5:** The performance of four rivals compared to FLUSS

	Autoplait_Classic_	Autoplait_Adapted_	HOG_1D_	Best human	Random
Win | lose | draw over FLUSS	3 | 26 | 3	3 | 25 | 4	8 | 15 | 9	11 | 9 | 12	0 | 32 | 0

A post-mortem analysis showed that if we had instead chosen between ¼ and ½ a period length, we would have cut the number of wins by all rivals by *more than half*. Nevertheless, these results strongly support our claim of the superiority of FLUSS.

### Robustness of FLUSS to the only parameter choice

**Fig. 18:**
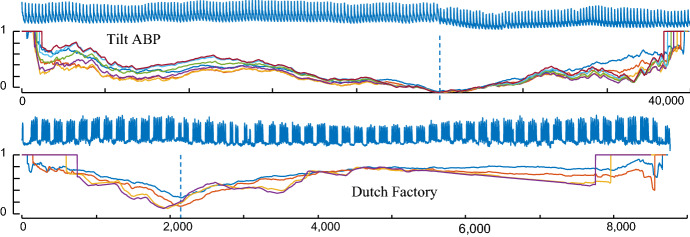
The CAC computed for (*top*) TiltABP with L = {100, 150, 200, 250, 300, 350, 400} and (*bottom*) DutchFactory for *L* = {25, 50, 200, 250}. Even for this vast range of values for *L*, the output of FLUSS is essentially unchanged (Color figure online)

The performance of FLUSS is highly robust to the choice of its only parameter, the subsequence length *L*. To demonstrate this, we consider two random datasets, *TiltABP* and *DutchFactory*, changing the subsequence length to span an order of magnitude, from 100 to 400 in TiltABP and from 25 to 250 in DutchFactory. As shown in Fig. 18, this variation of subsequence length has insignificant effect on the segmentation.

These results generalize to the remaining 30 datasets. To see this, we did the following experiments. For *all* thirty-two datasets we reran the experiments in the previous section, after *doubling* the subsequence length, and measuring change on our scoring function. Recall that because our scoring function is fine-grained, we only count a method’s success as *differing* if its score was at less than half, or more than double another score; otherwise, we report a *tie*.

Relative to the original experiment we found that for twenty datasets there was a tie, one got slightly better and eleven got slightly worse.

We then repeated the experiment, this time *halving* the subsequence length. This time, relative to the original experiment we found that for eight datasets there was a tie, twelve got slightly better and twelve got slightly worse (the raw numbers are archived at (Keogh 2017)). These results strongly support our assertion, our algorithm is not sensitive to the subsequence length parameter.

### Segmentation of multi-dimensional data

In Sect. 3.4 we showed an example of how we can trivially extend our framework to the multi-dimensional case (Machné et al. 2017). Here we test this ability with some formal experiments.

Note that there are two issues here: Given a *D*-dimensional time series, we must:

1. Choose which subset of *D*, *D*_sub_ to use as input to the segmentation algorithm. Note the *D*_sub_ may be as large as all *D* dimensions, or as few as one. However, work in the related problems of time series clustering and classification suggest that it will be rare that all *D* dimensions are useful (Hu et al. 2016).
2. Combine the results of the *D*_sub_ dimensions into a single segmentation prediction.

In this work, we gloss over the first issue, and assume that it is known, either from domain knowledge, or by learning it on snippets of labeled data.

**Fig. 19:**
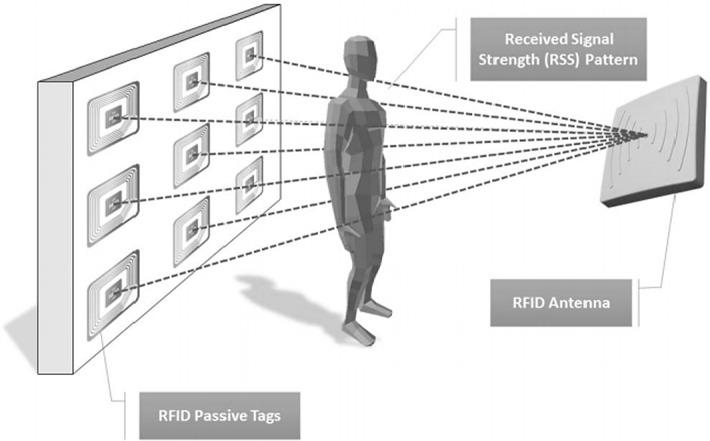
A passive RFID tags apparatus for posture recognition that eliminates the need for the monitored subjects to wear any devices. Because the line-of-sight between the RFID tags and the RFID antenna are at different heights, difference signals can (weakly) be seen as “experts” in providing information about the participants legs, torso and head Adapted from Yao et al. (2015)

We consider the Received Signal Strength Indicator (RSSI) dataset from (Yao et al. 2015). In this dataset, twenty-two activities for each of twelve time series from RFID data are provided. Figure 19 illustrates how the data was collected. It is obvious from this illustration that different time series have a different “view” of the participant. For example, some of the time series only register changes in the head/upper torso region.

**Fig. 20:**
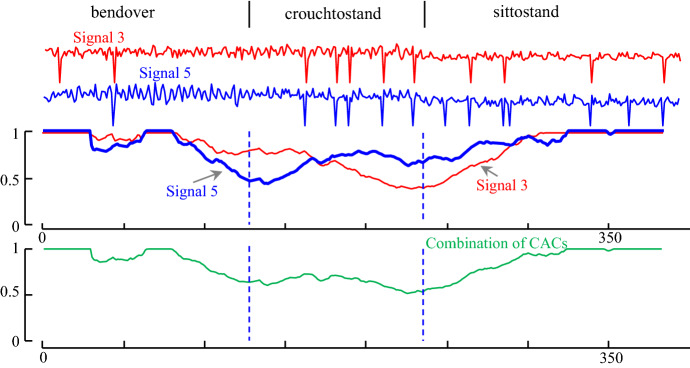
Multi-dimensional time series segmentation’s example on RSSI dataset-person 5. *top*) The CAC obtained from the signal number 3 (red) and the signal number 5 (blue) which each of them separately cannot be used for segmentation. *bottom*) the combination of two CACs which can segment all three activities (Color figure online)

We begin with a simple demonstration to illustrate the utility of incorporating multi-dimensional information into the segmentation process here. Segmentation of three activities on RSSI dataset is shown in Fig. 20 As we can see, Signal 5 is able to discover the transition from bendover to crouchtostand, but not the transition from crouchtostand to sittostand. In contrast, Signal 3 does not allow the discovery of the transition from bendover to crouchtoStand but does easily find the change from crouchtostand to sittostand.

Gratifyingly, as shown in Fig. 20.*bottom*, using both dimensions helps us find a more accurate segmentations than using *either* of the single dimensions.

**Fig. 21:**
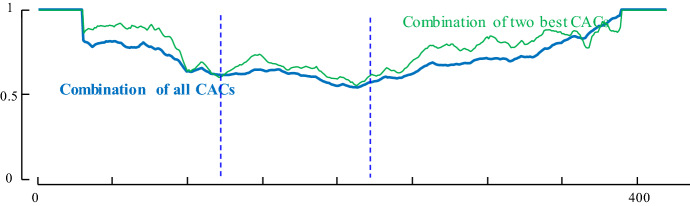
An example of our multi-dimensional time series segmentation on the three activities of RSSI dataset: bendover, crouchtostand and sittostand. The combination of CAC of two selected best time series (blue). The combination of all time series’CAC (green) (Color figure online)

In addition, to see how effective using all dimensions can be for segmentation, we calculate the score using *all* time series to segment the two activities. As it is shown in Fig. 21 the combination of all CAC’s “blurs” the resulting CAC somewhat.

To test the effectiveness of using *D*_sub_ dimensions in segmentation, we performed the following experiment. Two of the activities are selected randomly thirteen times and we calculate the CAC for combination of *all* time series and the two *best* time series. The result is compared with using just one time series, which has a best score for segmenting activities. In each run, we select two time series which have the best result in comparison to other two combinations and obtain the score of them. A time series counts as *winning* if its score is less than the score of best time series’ score. We report a *tie* when the score of two time series is equal. Table 6 summarizes the outcomes.

**Table 6:** The performance of all CACs and two of best CACs vs. only one time series

	Combination of all CAC’s	Combination of two time series’ CAC
Win | lose | draw over best CAC	1 | 11| 1	7 | 0 | 6

As shown in Table 6, in general, just using two (or some other *small* subset) of all the dimensions can segment the activities much more accurately than either using all dimensions or a single dimension. This can be seen as a classic “goldilocks” observation, and a similar observation is made in the context of time series classification in (Hu et al. 2016). This begs the question of *which* small subset of dimensions to use. We leave such considerations for future work.

**Fig. 22:**
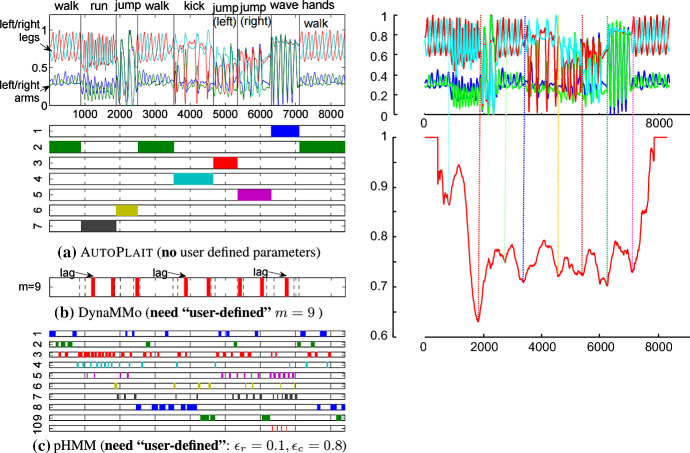
(Left-panel) A figure taken from (Matsubara et al. 2014a). The original caption reads “AUTOPLAIT can detect un-repeated regimes: **a** It captures all distinct motions (walking, running, etc.), while **b** DynaMMo was not completely successful in finding optimal cut points, **c** pHMM cannot find high-level motion patterns”. (right-panel) Our proposed algorithm achieves a segmentation that is also objectively correct and achieves a tying (near-perfect) score with AUTOPLAIT

As shown in Fig. 22 we also compare to the most cited multidimensional segmentation algorithm, Autoplait (Matsubara et al. 2014a).

As we noted in the previous section, we could not get the original Autoplait algorithm to produce any segmentation on 20 of our 32 single-dimensional test datasets. Recall that the algorithm was too *conservative* and did not produce *any* segmentation. We found this issue is even worse for the multidimensional segmentation setting. This is possibly because we are considering datasets for which the authors did not intend it to be applied to (although (Matsubara et al. 2014a) does not state any such limitations). Fortunately, we can bypass such issues. In Fig. 22.*left*-*panel* we show a screen capture of the original authors keystone multidimensional segmentation example. As this was a dataset was chosen by the authors to showcase their method, it is ideal for us to compare to. As the reader can see in Fig. 22.*left*-*panel*, our algorithm produces an equally successful segmentation. Moreover, the original authors use the example to compare to two other methods (DynaMMo and pHMM) they had invented and published in previous papers, showing that these methods failed on this example (Matsubara et al. 2014a). On this comparison our method and Autoplait tie. However, recall that we can segment such data in a streaming fashion, whereas Autoplait is batch algorithm only.

### The speed and utility of FLOSS

Our evaluation of FLOSS is brief, since it essentially inherits all the qualities of FLUSS but allows for *online* segmentation. However, to demonstrate the speed and utility of FLOSS, we performed the following experiment. We considered the Y-axis shoe acceleration from Subject 3 from the PAMAP dataset’s (Reiss and Stricker 2012) outdoor activity. The data is 270,000 data points sampled at 100 Hz, giving us 45 min of wall-clock time. We used FLOSS to maintain a sliding window of the last 20-s of the subject’s behavior, using a 65 subsequence length (suggested by (Reiss and Stricker 2012)). We discovered:

- It took us only 73.7 s to process the data; thus, we can process the data about 36 times faster than real time.
- In a post hoc sanity check, we examined the three lowest values of the CAC in this trace. By comparing the locations to the ground truth provided, we discovered that the Top-3 regimes changes we discovered correspond *exactly* to the following transitions: normal-walking|transient-activities, Nordic-walking*|*transient-activities and running|transient-activities.

**Fig. 23:**
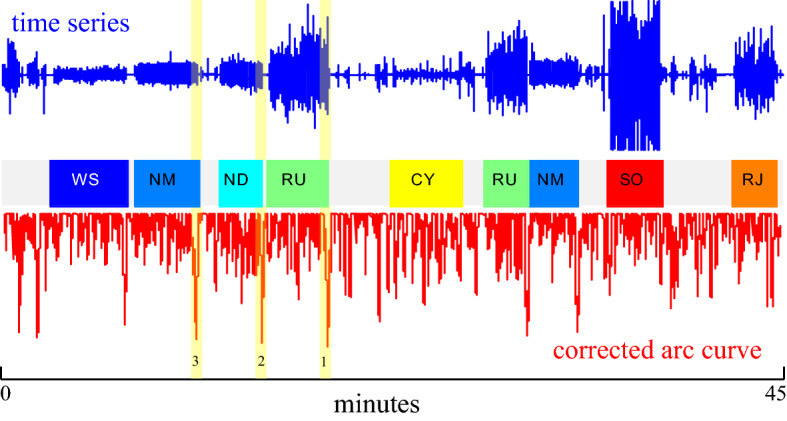
The CRC_1D_ (red) of a time series (blue) from PAMAP. The ground truth is shown in the center, where WS is walking slowly, NM is normal walking, ND is Nordic walking, RU is running, CY is cycling, RJ is rope jumping, and SO is playing soccer. The top three segmentation locations are marked with broad yellow translucent lines (Color figure online)

The output of FLOSS for this time series is shown in Fig. 23, and at (Keogh 2017), we have placed a video showing a trace of the process.

### Automatically setting FLUSS/FLOSS’s parameter

As discussed above, a great strength of FLUSS/FLOSS is that it has only one main parameter, the subsequence length *L*. Moreover, as we showed explicitly in Fig. 18, our algorithms are not particularly sensitive *L*’s value. Nevertheless, it is reasonable ask how one might go about setting this parameter, when faced with a novel domain.

While we will not exhaustively solve this issue here (it perhaps merits its own paper to do it full justice), we will show a simple heuristic that we empirically have found to be very effective. We consider our running example of Arterial Blood Pressure segmentation, which is shown in its entirety in Fig. 18.*top*.

Note that the problem reduces to a lack of labelled data. If we have some labeled data for the domain of interest, we can simply test all values of *L* and choose the one that minimizes our scoring function (Sect. 3.6). Our proposed heuristic is based on an idea that has been used for other data mining problems (Ha and Bunke 1997; Dau et al. 2016). If we only have snippets of a single class from our domain, but we also need labelled data that illustrates what the data looks like with a “distortion”, we can synthetically create such data by copying and then distorting our “clean” data. The success of this idea depends on our ability to produce a realistic distortion of the data. For example, in (Dau et al. 2016) the authors need to learn a parameter that is sensitive to time warping (local accelerations of the time series), so they introduce a function to artificially add warping to their limited training set. Here, the possible “distortions”, by definition, are unlimited and unknowable in advance. Although there are sophisticated methods (Esteban et al. 2017) for creating synthetic data, we simply used a change-of-rate by 5% as the regime change.

**Fig. 24:**
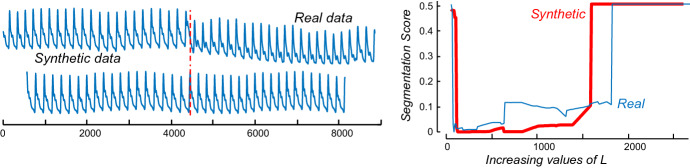
(Left) The real snippet of ABP data, with the regime change marked, and a snippet of ABP data (from the same individual) with a synthetic regime change created by appending the snippet to itself after rescaling it to 95% of its original length. (right) The *L* vs. score plots for both datasets a very similar, and more importantly, the value of *L* the minimized the score for the synthetic dataset also produces a very competitive value when applied to the real data

For concreteness, if *R* is our snippet of real data, then the following line of Matlab code Synthetic_Data=[R, R(1:0.95:end)]; produces our training data, and the correct location of the regime change should be at |*R*|. As Fig. 24.*left* shows, such a change is barely perceptible.

For the real data, the optimal value of *L* is 43, and this gives us a near perfect score of 0.0014. For our synthetic data, the optimal value of *L* is 68. If we had used this predicted value on the real dataset, the score would have been 0.0151. This is about equivalent to missing the regime change by plus or minus one half of a heartbeat.

An additional observation of this experiment is that it reinforces that claim that the algorithm is not too sensitive to the parameter length, any value of *L* from 20 to 590 would have discovered the correct location of the regime change within the length of a single heartbeat.

### A detailed case study in segmenting physical activity

In this section, we consider a case study that requires all three of the novel elements of this work. In particular, the *temporal arc constraint* (Sect. 3.5), the generalization to *multi*-*dimensional segmentation* (Sect. 4.7) and *learning the best subsequence length* from unsupervised data (Sect. 4.9).

Accurate measurement of physical activity in youth is extremely important as this behavior plays an important role in the prevention and treatment of obesity, cardiovascular disease, and other chronic diseases (Kozey-Keadle et al. 2011; Cain et al. 2013; Mu et al. 2013; Crouter et al. 2015). The use of wearables (i.e. accelerometers mounted on the wrist or ankle) reduce *recall*-*bias* common with questionnaires, but they do not provide contextual information needed by health care workers (Cain et al. 2013). Current methods use to map accelerometer data to physical activity outcomes rely on static regression models that have been shown to have poor individual accuracy during free-living measurements (Lyden et al. 2014). Recently, several research groups have recognized that *segmentation* of the data can be used as preprocessing step to improve accuracy of behavior classification (Cain et al. 2013; Crouter et al. 2015). This observation has an apparent *chicken*-*and*-*egg* nature to it, as many segmentation algorithms require at least much model-building, domain knowledge and parameter tuning, as the classification method they could potentially benefit (Lan and Sun 2015; Lin et al. 2016). However, as we have argued in this work, our proposed segmentation method is domain independent and only requires a single intuitive parameter to be set or learned.

The dataset we consider is the first “sanity check” dataset collected as part of a large-scale five-year NIH-funded project at the University of Tennessee Knoxville. Eventually, more than one hundred youth will be measured during a semi-structured simulated free-living period (development group) and one hundred youth will be measured during true free-living activity during an after-school program and at home (validation group). The need to segment this massive archive was one of the major motivations to develop FLOSS. Our initial dataset contains ten activities from a hip mounted sensor, including accelerometer and gyroscope data which is collected at 90 Hz. The data contains repeated activities, and both a visual inspection of the data and discussions with the domain experts that collected it strongly suggest that using just one dimension is not sufficient to meaningfully segment the activities.

In addition, we do not have a good intuition for what is an appropriate value for parameter *L* in this domain. However, as described in Sect. 4.9, we can estimate the best value of *L* using synthetic data. Our goal is to autonomously segment a time series containing multiple activities types with repetition into separate behaviors.

**Fig. 25:**
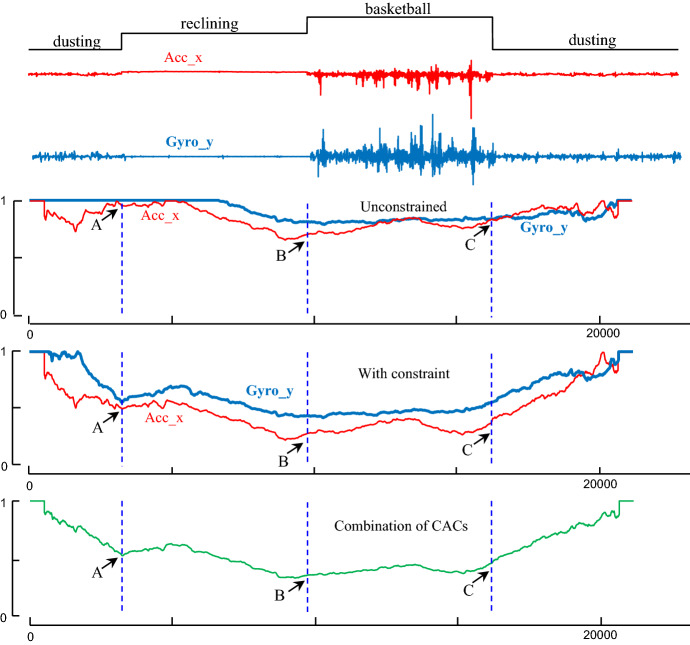
(Top to bottom) Ground truth segmentation, into dusting, reclining, basketball and dusting (gray). The accelerometer’s X (red) and the gyroscope’s Y (blue) data of hip from dataset provided by project (Crouter et al.). The classic CAC produces false negatives at locations A in both CAC. By applying arc constraint, gyroscope’s Y has detected the segment in location A, and accelerometer’s X data can detect the segments in location B and C. Combining two dimensions of data accelerometer’s X and gyroscope’s Y and considering all the properties can help us to detect all the segments in time series (Color figure online)

To illustrate this goal, we perform an experiment on a time series from dataset (Crouter et al.). As shown in Fig. 25, we considered four sequences of activities, dusting, reclining, basketball and (a return to) dusting. This figure clearly shows using one-dimension of data is not sufficient to segment the data, and not considering the possibility of repeated but disconnected activities misleads the algorithm. However, by addressing these issues produces more accurate results.

**Fig. 26:**
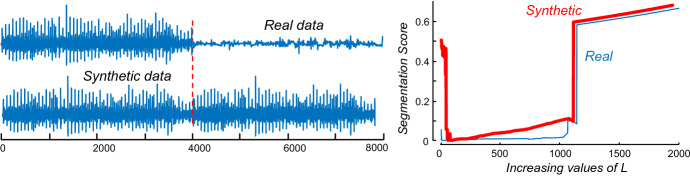
(Left) The real snippet of activity data, with the regime change marked, and a snippet of activity data with a synthetic regime change created by appending the snippet to itself after rescaling it to 95% of its original length. (right) The *L* vs. score plots for both datasets. The value of *L* that minimized the score for the synthetic real dataset is very similar

In producing Fig. 25, for simplicity we hardcode the *L* value. However, we can also learn the value of subsequence length by using synthetic data from just one randomly chosen class, in this case walking slow, and applying 5% rescaling trick discussed in Sect. 4.9. We evaluate our prediction of the best value for *L* by comparing the results on real data from segmenting two activities of walking slow and catch. As shown in Fig. 26 the subsequence length is predicted from synthetic data is very similar to value obtained with real data.

As it is shown in Fig. 26. *right*, the value of *L* which minimizes the score function in both the synthetic proxy data and ground truth data is about 100 (about one second). The experiments in Figs. 25 and 26 strongly suggest that for segmenting real-world data, all the elements proposed in this paper is necessary.

## Summary and future work

We have introduced a fast, domain-independent, online segmentation algorithm and have shown its utility and its versatility by applying it to dozens of diverse datasets.

A limitation of our algorithm is that it requires setting a parameter. However, we demonstrate that our algorithm is insensitive to the value of this parameter. Moreover, we show that in at least some cases, it can be learned directly from unlabeled data from the same domain. Another limitation of our algorithm is that is assumes that each regime will manifest with at least two repeated periods.

We have further shown that our algorithm also works for the multi-dimensional case (Keogh 2017), and allows the user to specify a domain dependent temporal constraint, to allow segmentation of shorter repeated regimes set within longer period repetitions.

We have made all code and data freely available to the community to confirm, extend, and exploit our work. For future work, we are interested in applications of our ideas, for example, to learning from weakly labeled-data (Hao et al. 2013), and to time series summarization and visualization.

## Footnotes

1 We only considered a subset of 12 from the full dataset to be respectful of the participant’s time and attention.
